# Essential and Potentially Toxic Elements (PTEs) Content in European Tea (*Camellia sinensis*) Leaves: Risk Assessment for Consumers

**DOI:** 10.3390/molecules28093802

**Published:** 2023-04-28

**Authors:** Federico Girolametti, Anna Annibaldi, Silvia Illuminati, Elisabetta Damiani, Patricia Carloni, Cristina Truzzi

**Affiliations:** 1 Department of Life and Environmental Sciences, Università Politecnica delle Marche, 60131 Ancona, Italy; f.girolametti@staff.univpm.it (F.G.);; 2 Department of Agricultural, Food and Environmental Sciences, Università Politecnica delle Marche, 60131 Ancona, Italy

**Keywords:** tea, *C. sinensis*, leaves, essential elements, potentially toxic elements (PTEs), Europe, food quality, risk assessment, food security

## Abstract

Tea (*Camellia sinensis*) is the second most consumed beverage worldwide, playing a key role in the human diet. Tea is considered a healthy drink, as its consumption has been linked to a lower risk of cardiovascular disease-related events and death, stroke, metabolic syndrome and obesity. However, several studies have shown that *C. sinensis* is a hyperaccumulator of Al and other elements that are considered potentially toxic. In the present study, the contents of 15 elements (both essential and toxic) were determined for the first time in tea leaves collected in tea gardens located in six different European countries and processed to provide black and green tea. The results showed that Al was the major toxic element detected, followed by Ni, Cr, Pb, As, Cd, Ag, and Hg. Essential elements were detected in the order of Mn, Fe, Zn, Cu, Co, and Se. Statistically significant correlations (*p* < 0.05) were found in the distribution of some elements, highlighting mechanisms of synergic or antagonist interaction. Multivariate analysis revealed that geographical origin was the main driver in clustering the samples, while the different treatment processes (black or green) did not significantly affect the contents of elements in the leaves. The estimation of potential non-carcinogenic risk revealed no risk for the consumption of European teas for consumers in terms of potentially toxic elements.

## 1. Introduction

Tea (*Camellia sinensis*) is a perennial evergreen plant of which two main varieties exist for tea production: *Camellia sinensis var. sinensis* and *Camellia sinensis var. assamica* [1]. The first evidence of the use of this plant to produce infusions dates back to the III century AD (China). At the beginning of the XVII century, it was imported into Europe, and in a few decades, its trade expanded throughout the continent. Today, according to the FAO [2], the annual global tea production amounts to over USD 17 billion and the world tea trade is valued at about USD 9.5 billion. Production is projected to increase annually by 2.1% (black tea) and 6.3% (green tea) by 2030 [2]. Tea is generally divided into categories based on the processing methods that the leaves undergo after harvesting, such as white, yellow, green, oolong, black, and post-fermented (dark). Tea is the second most consumed beverage in the world (after water), and it is particularly appreciated by consumers both for its flavor and its multiple beneficial properties. Numerous studies have demonstrated how an appropriate consumption of tea is related to a reduction in serum cholesterol, prevention of low-density lipoprotein oxidation, and decreased risk of cardiovascular disease and cancer [3]. This is due to the presence of various chemical compounds, such as polyphenols, fluorides, caffeine, and essential minerals, that exert positive effects on the human body [4]. However, not all the elements that can be accumulated in tea leaves are essential. Several studies have shown that *C. sinensis* is an Al hyperaccumulator [5], accumulating both Al and other elements that are considered potentially toxic for humans (e.g., Hg, Pb, Cd, and As). These contaminants have both natural and anthropogenic origins, but their release into the environment is increased by several human activities such as mine exploitation, melting and other industrial and agricultural processes [6]. The accumulation of these elements in tea plants depends on various environmental factors, such as the chemical composition of the soil in terms of element content, pH, and atmospheric depositions [7,8]. It has been demonstrated that a certain percentage of these elements are transferred to the liquid phase during the infusion process, and this transfer ratio depends on the time of infusion and water temperature [9,10]. To date, the European community has not provided threshold values for the contents of these elements in tea leaf or infusion, since the legislation that regulates these values refers to foodstuffs such as fresh herbs and leaf vegetables [11]. However, the United States Environmental Protection Agency (US EPA) and the World Health Organization (Joint FAO/WHO Expert Committee on Food Additives) provide recommended doses not to be exceeded for some elements. Although several reviews and studies on the element contents of tea leaves have been conducted in Asia and Africa [12,13,14,15,16,17,18,19,20,21,22,23], to the best of our knowledge, no studies have been performed on tea samples from Europe. The only two carried out studies referred to a local market in southern Poland and a tea house located in Slovakia, but none of the leaves collected in these studies came from Europe [24,25]. The present study was aimed to deepen the knowledge of the chemical composition of tea leaves collected from different gardens located in six European countries (Italy, Switzerland, the United Kingdom, the Netherlands, Germany and Portugal) and processed to provide black and green teas, to assess their state of contamination, and to investigate how factors such as treatment process or geographical origin may influence the different accumulation levels of these elements. Moreover, the health risk of exposure to these elements by European consumers was evaluated with hazard quotient (HQ) and hazard index (HI) estimations.

## 2. Results

**Figure 1:**
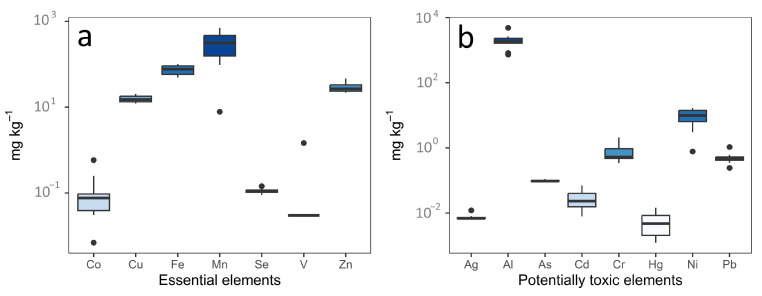
Essential (**a**) and potentially toxic (**b**) element contents in European tea leaves.

Figure 1 shows the mean contents (mg kg^−1^) of essential and potentially toxic elements in European tea.

### 2.1. Essential Elements

The average levels of essential elements are arranged according to the following order: Mn > Fe > Zn > Cu > V > Co > Se (Figure 1a and Table 1).

The element with the highest content in the tea leaves was manganese (Mn), with a mean content of 322 ± 214 mg kg^−1^. The highest values were recorded in the green and black teas of the Korean cultivar from Germany (709 ± 9 and 628 ± 5 mg kg^−1^, respectively), and the lowest concentration was registered in the green tea from Portugal (8 ± 1 mg kg^−1^). Statistically significant differences (*p* < 0.0001) were recorded among almost all the samples. Iron (Fe), the second most abundant element, showed an overall concentration of 76 ± 19 mg kg^−1^, with the highest value recorded in the black tea from the Netherlands (101 ± 2 mg kg^−1^), and the lowest value detected in the black tea of the Korean cultivar from Germany (49 ± 1 mg kg^−1^), with statistically significant differences among many samples (*p* < 0.0001). For zinc (Zn), an average content of 29 ± 7 mg kg^−1^ was detected, with the significantly highest value recorded in the black tea from the United Kingdom (46±1 mg kg^−1^) with respect the other samples (*p* < 0.0001). The significantly lowest values were found in the black tea of the Vietnam and Azores cultivars from Germany (21.9 ± 0.2 and 22 ± 1 mg kg^−1^, respectively) and Italy (22.6 ± 0.2 mg kg^−1^) (*p* < 0.0001). Most of the samples showed statistically significant differences in Zn content with respect to the other samples. The content of copper (Cu) was also characterized by statistically significant differences among samples (*p* < 0.0001), with a mean value of 16 ± 3 mg kg^−1^, from 12.2 ± 0.3 mg kg^−1^ in the black tea of the Korean cultivar from Germany to 20.3 ± 0.7 mg kg^−1^ of the green tea from Italy. The vanadium (V) content was always below the LOQ (0.06 mg kg^−1^), except for green tea leaves from the Netherlands (1.5 ± 0.2 mg kg^−1^). The mean content of selenium (Se) was 0.11 ± 0.02 mg kg^−1^, and statistically significant higher contents were found in the green tea from Portugal and the black tea of the Vietnam cultivar from Germany (0.14 ± 0.02 mg kg^−1^ for both) with respect to the lowest values found in the black tea of the Azores and Korean cultivars from Germany (0.089 ± 0.006 and 0.09 ± 0.02 mg kg^−1^, respectively) (*p* = 0.007546). The mean recorded level of cobalt (Co) was 0.1 ± 0.2 mg kg^−1^, with the minimum content (0.007 ± 0.001 mg kg^−1^) and maximum content (0.581 ± 0.019 mg kg^−1^) in the green and black tea, respectively, from Italy, which were statistically different from each other and from the other samples (*p* < 0.0001).

### 2.2. Potentially Toxic Elements

The potentially toxic elements’ concentrations followed the order of Al > Ni > Cr > Pb > As > Cd > Ag > Hg (Figure 1b and Table 2).

The overall average aluminum (Al) content was 1986 ± 1086 mg kg^−1^, with the highest value registered in the green tea of the Korean cultivar from Germany (4865 ± 933 mg kg^−1^), which was significantly different (*p* < 0.0001) from the other samples; the lowest values were detected in the green tea from Portugal (733 ± 8 mg kg^−1^) and in tea samples from the Netherlands (green tea, 735 ± 5 mg kg^−1^; black tea, 815 ± 30 mg kg^−1^), which were statistically different from all the other samples (*p* < 0.0001). The mean concentration of nickel (Ni) was 10 ± 5 mg kg^−1^, and its maxima were recorded in the black and green teas from Switzerland (16.8 ± 0.5 and 16.7 ± 0.2 mg kg^−1^, respectively), which were significantly higher (*p* < 0.0001) than the Ni contents found in the other samples. The significantly lowest content was recorded in the green tea from Portugal (0.77 ± 0.02 mg kg^−1^), and statistically significant differences in Ni content were observed between different tea gardens. The mean chromium (Cr) content was 0.8 ± 0.5 mg kg^−1^, from 0.340 ± 0.005 mg kg^−1^ in the black tea of the Azores cultivar from Germany to 2.08 ± 0.01 mg kg^−1^ in the black tea from the United Kingdom, with statistically significant differences (*p* < 0.0001) among most of the samples. We recorded an average lead (Pb) level of 0.6 ± 0.2 mg kg^−1^, and the samples from the Netherlands were the most contaminated by this element (1.1 ± 0.1 and 1.06 ± 0.07 mg kg^−1^), with statistically significant differences with respect to the other samples (*p* < 0.0001). The Pb content in teas from Germany of the Azores cultivar (0.24 ± 0.02 mg kg^−1^) was statistically lower than the Pb contents in the other samples (*p* < 0.0001), except for the green tea from the United Kingdom. Arsenic (As) showed a mean concentration of 0.098 ± 0.007 mg kg^−1^ (min–max, 0.090−0.111 mg kg^−1^), with no statistically significant differences among the samples (*p* = 0.5680). The mean concentration of cadmium (Cd) was 0.03 ± 0.02 mg kg^−1^, with the green tea from the Netherlands (0.061 ± 0.002 mg kg^−1^) and the black tea of the Azores cultivar from Germany (0.071 ± 0.004 mg kg^−1^) being the most contaminated and showing statistically higher (*p* = 0.0002) contents than the other tea leaves. The green tea from Portugal showed a Cd content (0.008 ± 0.0003 mg kg^−1^) that was statistically lower than that of the other teas (*p* = 0.0002); moreover, almost all the samples showed statistically significant differences in Cd content (*p* = 0.0002). The presence of silver (Ag) was also detected, with a mean value of 0.007 ± 0.001 mg kg^−1^. The green tea from Switzerland showed the highest value (0.0120 ± 0.0004 mg kg^−1^), which was statistically higher (*p* < 0.0001) than that of the other samples. The green tea from Portugal (0.0064 ± 0.0001 mg kg^−1^) and the black tea of the Vietnam cultivar from Germany (0.0064 ± 0.0001 mg kg^−1^), showed statistically lower Ag contents with respect to the black tea from the United Kingdom and the Netherlands, as well as the green tea from the United Kingdom and Switzerland (*p* < 0.0001). The average content of mercury (Hg) was 0.006 ± 0.004 mg kg^−1^, and the green tea of the Korean cultivar from Germany was the most contaminated (0.0145 ± 0.0001 mg kg^−1^), with a statistically higher (*p* < 0.0001) content compared with the other samples. The green tea from the United Kingdom and the black tea from Switzerland (0.0013 ± 0.0001 and 0.0012 ± 0.0001 mg kg^−1^, respectively) showed the lowest values, which were significantly lower than those of the other samples, except for the green teas from Italy, the Netherlands, and Portugal.

### 2.3. Black vs. Green Tea

**Figure 2:**
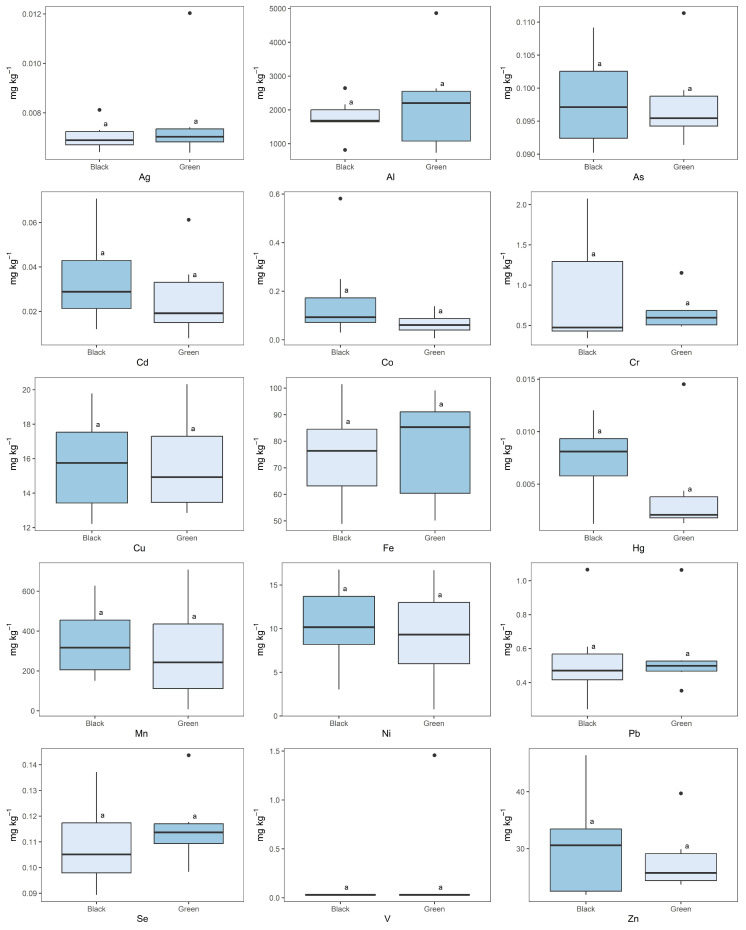
Comparison between element contents in the black and green European tea leaves. Different letters within the same plot indicate statistically significant differences in element contents between the two different types of tea (*p* < 0.05).

In the present study, tea leaves were divided in two groups, black and green, to investigate the possible influence of the treatment process on the content of the studied elements. Due to the high variability in the content of elements in leaves coming from different parts of Europe, no statistically significant differences (*p* > 0.05) in the element contents between the two types of leaves were found (Figure 2). The overall mean contents of elements in black vs. green tea were: Ag, 0.0070 ± 0.0006 vs. 0.008 ± 0.002 mg kg^−1^; Al, 1778 ± 560 vs. 2230 ± 1524 mg kg^−1^; As, 0.098 ± 0.007 vs. 0.098 ± 0.007 mg kg^−1^; Cd, 0.03 ± 0.02 vs. 0.03 ± 0.02 mg kg^−1^; Co, 0.2 ± 0.2 vs. 0.07 ± 0.05 mg kg^−1^; Cr, 0.9 ± 0.7 vs. 0.7 ± 0.3 mg kg^−1^; Cu, 16 ± 3 vs. 16 ± 3 mg kg^−1^; Fe, 75 ± 18 vs. 78 ± 21 mg kg^−1^; Hg, 0.007 ± 0.004 vs. 0.004 ± 0.005 mg kg^−1^; Mn, 345 ± 181 vs. 295 ± 262 mg kg^−1^; Ni, 11 ± 5 vs. 9 ± 6 mg kg^−1^; Pb, 0.5 ± 0.3 vs. 0.6 ± 0.3 mg kg^−1^; Se, 0.11 ± 0.02 vs. 0.12 ± 0.02 mg kg^−1^; and Zn, 30 ± 9 vs. 28 ± 6 mg kg^−1^.

However, considering the black and green tea leaves that were processed in the same garden, some differences can be underlined. In Jersey Fine Tea, the contents of the essential elements Co, Cu, Fe, Mn were statistically higher (*p* = 0.0020, *p* = 0.0101, *p* = 0.0013 and *p* < 0.0001, respectively) in the green tea leaves compared with the black leaves, except for Zn, which showed the opposite pattern (*p* = 0.0007). The concentrations of the toxic elements Cr, Hg, and Ni were statistically higher (*p* < 0.0001, *p* = 0.0058, and *p* = 0.0001, respectively) in the black tea leaves. In the Swiss garden, Cu, Mn, Zn, and Cd were present in statistically higher (*p* = 0.0008, *p* = 0.0003, *p* < 0.0001 and *p* = 0.0284) levels in the black tea leaves, while Ag, Cr, and Hg were more concentrated (*p* < 0.0001, *p* = 0.0004 and *p* = 0.0159, respectively) in the green tea leaves. The green tea leaves from the garden located in Italy showed a statistically higher content of Cu (*p* = 0.0011), Mn (*p* < 0.0001) and Zn (*p* = 0.0002) than the corresponding black tea leaves, while the concentrations of Co, Cr, and Hg were higher (*p* = 0.0003, *p* < 0.0001 and *p* = 0.0002, respectively) in black tea leaves. In the Het Zuyderblad garden, statistical differences were recorded between the two types of tea leaves, as the V (*p* = 0.0001), Cd (*p* < 0.0001) and Ni (*p* < 0.0001) contents were higher in the green leaves while the Cu, Mn, Cr and Hg contents were more concentrated (*p* = 0.0001, *p* = 0.0063, *p* < 0.0001 and *p* = 0.0049, respectively) in the black leaves. Finally, the Korean cultivar from the German garden showed statistically higher contents of Co (*p* = 0.0010), Mn (*p* = 0.0002) and Zn (*p* < 0.0001) in the black tea leaves with respect to the green leaves.

### 2.4. Multivariate Analysis

**Figure 3:**
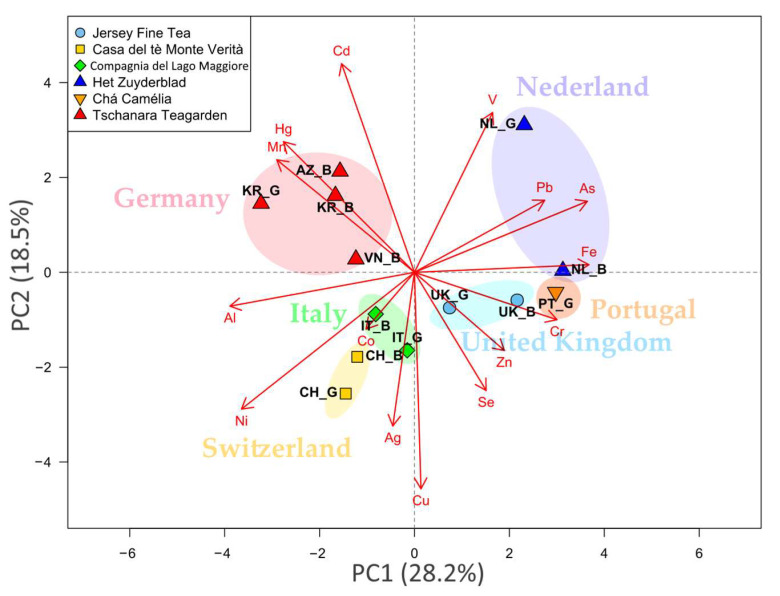
2D biplot of PC1 vs. PC2. B: black tea; G: green tea; CH: Switzerland; PT: Portugal; NL: the Netherlands; IT: Italy; UK: the United Kingdom; KR: the Republic of Korea; VN: Vietnam; AZ: Azores.

A principal component analysis (PCA) was applied to the dataset in order to reduce the dimensionality of the data matrix and to enable the easier visualization of the results for data interpretation (Figure 3). The PCA extracted five significant, cross-validated principal components that accounted for 81.2% of the variability in the original data (Table S1).

The first two PCs (cumulative variance of 46.7%) showed that samples collected in the same garden were graphically close together due to similarities in element composition. Samples from Tschanara Teagarden (Odenthal, Germany) were characterized by higher contents of Mn, Hg and Cd with respect to the other samples; tea leaves from the Het Zuyderblad garden (Soerendonk, the Netherlands) showed higher contents of V, Pb, As and Fe; Jersey Fine Tea (Jersey, UK) and Chá Camélia (Fornelo, Portugal) samples showed higher contents of Cr, Zn, and Se with respect to the other samples. Finally, tea leaves coming from the Italian and Swiss gardens were close to each other, showing higher contents of Al, Ni and Ag with respect to the other samples. In fact, these two gardens are geographically very close, at only 40 km apart. Like the first two PCs, PC3 separated the samples according to the garden of origin. Finally, PC4 and PC5 did not seem to show any particular clustering with regard to leaf processing or geographical origin (Figure S1).

**Figure 4:**
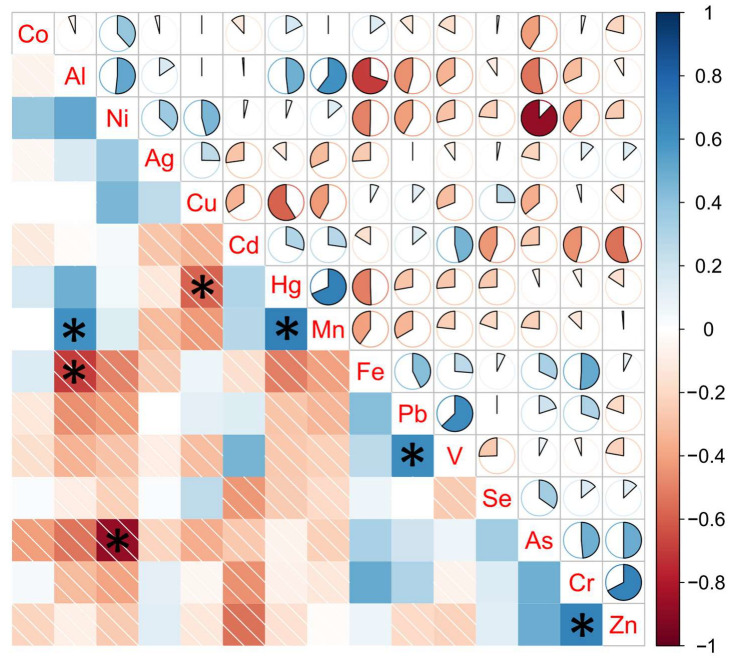
Correlogram plot on element content in European tea leaves. * denotes a statistically significant correlation (*p* < 0.05).

Additionally, a correlation analysis was performed to identify possible correlations among element contents. Figure 4 shows the correlogram generated with the element contents of European tea leaves.

This analysis highlighted statistically significant correlations (*p* < 0.05) between the pairs of Al–Mn (*p* = 0.0292), Cr–Zn (*p* = 0.0118), Hg–Mn (*p* = 0.0097), and Pb–V (*p* = 0.0215) (positively correlated) and the pairs of Al–Fe (*p* = 0.0080), As–Ni (*p* = 0.0001), and Cu–Hg (*p* = 0.0325) (negatively correlated).

### 2.5. Estimation of Hazard Quotient (HQ) and Hazard Index (HI)

The results of the hazard quotient (HQ) and hazard index (HI) are shown in Table 3. HQs were always less than 1, and Al and Mn were the most representative elements, with HQs of 4.8 × 10^−2^ and 2.2 × 10^−2^, respectively. The HI ranged between 2.1 × 10^−2^ (Chá Camèlia, Fornelo, Portugal) and 1.1 × 10^−1^ (Tschanara Teagarden, Odenthal, Germany). The overall HI mean value was 7.5 × 10^−2^, indicating no significant, non-carcinogenic health hazard for European consumers in the consumption of black and green tea originating from European tea gardens.

## 3. Discussion

The chemical composition of tea leaves has been investigated in multiple studies, mainly regarding tea plants grown in Asia and Africa. It is known from the literature that the element contents in tea leaves mainly depends on the root uptake capacity, the element content in the soil, and the soil’s acidification. Atmospheric depositions may even contribute to the accumulation of specific elements [26]. Therefore, some differences in element composition between tea leaves coming from different regions was expected. Our results were sometimes consistent with literature data, within the range of experimental error, and sometimes showed different orders of magnitude in terms of element content.

### 3.1. Essential Elements in Tea Leaves

In this work, the overall content of essential elements in decreasing order was: Mn > Fe > Zn > Cu > V > Co > Se. This trend was in agreement with the contents found in medicinal and aromatic plants originating from Europe and the Mediterranean region, where the contents of beneficial essential elements commonly decreases from Fe and Mn to Zn and Cu [27]. For a comparison with literature data, we report the average concentrations of the essential elements detected in tea leaves from different countries in Table 4.

Manganese (Mn) is an essential element that is involved in the synthesis and activation of many enzymes and the regulation of the metabolism of glucose and lipids in humans [28]. As for Fe, excessive Mn exposure could increase the generation of reactive oxygen species (ROS) and result in oxidative stress [29]. Mn(II) is the only plant-available form that can be transported into the roots and translocated to the shoot [30]. The Mn contents in tea leaves from this study showed lower values than teas from other countries [14,16,17,19,20], except for herbal teas from Egypt [17], compared with which they showed higher values. The contents of this element in European tea leaves were higher than the values recorded in most commonly consumed medical plants, but it is known that tea has more Mn-rich leaves compared with other plants [27].

Iron (Fe) is an essential constituent of hemoproteins and iron–sulfur proteins, and its activities include DNA synthesis, cell metabolism, and oxygen transport [31]. However, because it is a Fenton metal, excessive Fe concentrations can cause oxidative stress in biological tissue [32]. Fe uptake can be reduced by stimulating the growth of tea plants with an Al supply [33]. The Fe contents found in the studied European tea were similar to the concentrations recorded in tea from Ethiopia [19]; lower than those in tea from Egypt, Iran, India, and Ceylon [17]; and higher than those in tea from Taiwan [18]. Most medicinal and aromatic plants have a leaf Fe level in the range of 50–100 mg kg^−1^, which is the critical level below which deficiency symptoms develop in plants [27].

Zinc (Zn) is one of the major essential elements, and its deficiency has been associated with several diseases [34]. It is a naturally occurring element in soils, but its content may exceed normal values when Zn-contaminated fertilizers, pesticides or sewage sludge are used [35]. Its uptake ratio from soil depends on phosphorus bioavailability since both nutrients show antagonistic behavior in plant nutrition [36]. In the studied European teas, the contents of Zn were similar to those found in other studies, except for Taiwanese tea.

Copper (Cu) is a micronutrient for plants, but it can become toxic if present in high concentrations [37]. It is one of the native metals found in tea, playing a key role in polyphenol oxidase enzymes [5]. Except from Taiwanese tea, the values found in this study perfectly fit with those found in the literature [13,14,16,17,19,20,22,23] and fell within the range of 5–20 mg kg^−1^ normally required for plant growth; levels of higher than 30 mg kg^−1^ are unhealthy for plants [27].

Vanadium (V) is an essential element in most living beings [38]. The content of this element has been investigated in very few studies. The V contents in our tea samples were slightly lower than those of tea leaves from China (0.49–1.58 mg kg^−1^) [39,40] but similar to those of commercially available herbal teas from Brazil, where the highest content was recorded in lemongrass tea [41]. Our samples contained lower V contents with respect to herbal plants from Turkey, which can contain up to 20 mg kg^−1^ of this element [27].

Cobalt (Co) is an essential element necessary for the formation of vitamin B_12_ (hydroxocobalamin) [42]. The Co contents in tea leaves from this study were consistent with the literature, where this element has generally been found to be present in concentrations of less than 1 mg kg^−1^ [18,19,23]. A comparison between the levels of this element in the studied European tea leaves and those found in other medical plants revealed that the Co contents were similar, as the range of variability in medical herbs is from 0.05 to 1.76 mg kg^−1^ [27].

Selenium (Se) is an essential element because it protects the integrity of cell membranes, and its coenzymatic role in the metabolism of thyroid hormones has been demonstrated [43]. It is transported from soil to plants through sulfate transporters present in the plasma membrane of root cells. Se is assimilated as organic Se via sulfur metabolism and volatilized as dimethylselenide (DMSe) and dimethyldiselenide (DMDSe) into the atmosphere. However, to date, very few studies have investigated Se contents in tea leaves. The results presented here are similar to those recorded in Taiwan [18]. Among other similar products, spices and herbs from Turkey can contain up to 5 mg kg^−1^ of Se, a much higher concentration than the typical content in tea leaves [27].

**Table 4:** Essential element contents in tea leaves expressed as mean ± standard deviation (min–max) in mg kg^−1^.

Garden or Market Location, Country	Type	Co	Cu	Fe	Mn	Se	V	Zn	Reference
European Gardens	Black	0.2 ± 0.2 (0.03–0.58)	16 ± 3 (12.2–19.8)	75 ± 18 (48.9–101)	345 ± 181 (150–628)	0.11 ± 0.02 (0.089–0.14)	<0.03	30 ± 9 (21.9–46.4)	This study
European Gardens	Green	0.07 ± 0.05 (0.01–0.14)	16 ± 3 (12.9–20.3)	78 ± 21 (50.1–99.2)	295 ± 262 (7.8–709)	0.12 ± 0.02 0.098–0.12)	0.3 ± 0.6 (<0.03–1.46)	28 ± 6 (23.7–39.7)	This study
Sylhet and Moulvibazar district, Bangladesh	Black					(0.006–10.8)			[12]
India	Black		14.56 ± 6.85 (0.033–52.26)						[13]
Sri Lanka	Black		11.29 ± 5.906 (0.033–64–24)						[13]
Cairo, Egypt	Black	<LOQ	17.3 ± 1.7	213.3 ± 47.1	809.0 ± 288.3			26.7 ± 3.5	[14]
Cairo, Egypt	Green	<LOQ	18.5 ± 4.52	218.5 ± 18.62	991.8 ± 66.2			31.86 ± 6.72	[14]
Cairo, Egypt	Herbal	<LOQ	15.6 ± 1.63	373.5 ± 198	52.3 ± 2.03			24.7 ± 9.4	[14]
Local market, China	Green	0.29 ± 0.13 (0.11–0.58)	17.04 ± 4.69 (8.42–31.48)						[23]
Anhui, China	8 varieties		24.21 ± 5.22 (17.23–40.01)		1293.71 ± 696.25 (581.16–2844.22)				[16]
Gilan, Iran	Black		49.39	188.1	608.3			24.10	[17]
India	Black		23.21	146.9	496.3			27.26	[17]
Ceylon	Black		38.16	168.14	488.8			28.31	[17]
Hsinchu market, Taiwan	Green	0.7 (0.4–1.2)	0.4 (0.2–0.9)	0.6 (0.4–1.2)		0.3 (0.1–0.6)		6.3 (4.8–9.7)	[18]
Hsinchu market, Taiwan	Oolong	0.3 (0.09–0.6)	0.9 (0.7–1.5)	0.9 (0.7–1.3)		0.09 (0.005–0.1)		3.4 (2.5–4.6)	[18]
Hsinchu market, Taiwan	Black	0.2 (0.06–0.4)	0.3 (0.05–0.7)	0.9 (0.7–1.2)		0.06 (0.001–0.1)		1.2 (0.9–1.5)	[18]
Wushwush, Ethiopia	Green	(0.03–2.84)	(<LOD–19.15)	(26.03–100.43)	(501–1281)			(57.9–330.5)	[19]
Guizhou, China			10.70 ± 3.42 (6.17–16.25)		1940 ± 1120 (536–4610)			13.5 ± 2.6 (9.1–20)	[20]
Yunnan, China	Puerh		11.7 ± 1.7 (9.4–13.8)					12.1 ± 3.3 (7.7–17.5)	[22]
Local market, Brazil	Lemongrass						0.08 ± 0.028		[41]
Not specified	Black						1.58 ± 0.10		[39]
China	Different varieties						(0.49–0.55)		[40]

### 3.2. Potentially Toxic Elements in Tea Leaves

A comparison of the results of this study with the average concentrations of potentially toxic elements (PTEs) detected in tea leaves reported in the literature from different countries is shown in Table 5.

Food contamination with lead, mercury, cadmium, and arsenic is regulated by Commission Regulation (EC) No 1881/2006 and amending regulations [11,44], which set the maximum levels for certain contaminants in foodstuffs.

Among the most occurring contaminants in herbal plants, lead (Pb) and cadmium (Cd) are the most studied. Pb is one of the most dangerous because it can cause adverse effects even at very low concentrations [45]. The sources of contamination in tea plants are the uptake from the soil and the direct deposition from atmospheric particulate matter [46]. Pb accumulates in tea leaves, and its content in older leaves is higher than in younger ones [47]. For this element, the results of our study were lower than those found in China [15,16,17,20,22,23], and they were slightly higher than concentrations recorded in India, Egypt, and Taiwan [13,14,18]. Different surveys conducted on herbal plants have shown concentrations of Pb of the same order of magnitude or higher than 0.6 mg kg^1^ (the average content in European tea leaves from this study) [27,48,49]. For example, medicinal plants from Turkey and Egypt had contents of 0.7–1.7 mg kg^−1^ and 0.3–1.8 mg kg^−1^, respectively, whereas higher values of up to tens of mg kg^−1^ were found in some plants such as *Thymus serrulatus*, *Centella asiatica* and *Allium sativum*. Tea leaves are therefore among the least contaminated plants.

Cadmium (Cd) is a multi-tissue carcinogenic element [50], and its presence and accumulation in soil are mainly due to phosphate fertilizer application, which represents 50% of total input in agricultural land [51]. The availability of Cd for plants is influenced by several soil factors, such as the pH, humus, and organic carbon content [27]. This element occurs at µg kg^−1^ concentrations, as confirmed by other studies [12,13,14,18,19,20,23]. As for Pb, the contents of Cd in the studied European tea leaves (0.03 ± 0.02 mg kg^−1^) were among the lowest values compared with other commercially available herbal plants [27,48,49], which show concentrations of above 0.5 mg kg^−1^ in most cases.

Mercury (Hg) is one of the most dangerous pollutants. The MeHg form can bioaccumulate and biomagnify in the food chain, causing toxic effects on several organs, neurological damage, and a variety of diseases [52]. Its content in tea leaves mainly depends on two factors: soil conditions [7] and atmospheric depositions [8]. As for Pb, a positive correlation between leaf age and Hg content has been demonstrated [21]. The mean concentration recorded in this study was 0.007 mg kg^−1^, as found in other areas of the world [13,20,21]. The concentration of this element has been investigated in 120 herbal drugs, and the recorded values have been below 0.1 mg kg^−1^, as with our samples [27]. The very low Hg concentration in tea leaves, as well as in herbal plants in general, is due to its very low availability in plant samples [27].

Arsenic (As) is a mutagenic and carcinogenic element that exerts toxic effects on plants and animals [53]. However, tea does not show particular As accumulation mechanisms, and the content of this element is low if uncontaminated soils are used, depending on their bioavailability [54]. The As content in tea leaves from this study (~0.1 mg kg^−1^) was similar to that found in other studies [12,13,15,20]. In other plants, As is generally reported at concentrations that range from 0.2 to 1.2 mg kg^−1^ (*Salix* leaves, *Mentha spicata*, and fennel fruit) [27]. Various tea bags of widely consumed herbs in Bulgaria were reported to have 0.02–0.25 mg kg^−1^ of As, a range that fits with our results [27].

Silver (Ag) nanoparticles are globally applied because of their antimicrobial properties, and their increasing use is leading to major environment and human health exposure [55]. The ability of plants to interact, translocate and biotransform Ag is well known [56]. However, to the best of our knowledge, our study is the first to have determined the concentrations of Ag in tea leaves. In our samples, the Ag content is very low, in the order of µg kg^−1^. A study on infusions of different brands of Yerba Mate Tea in Brazil detected Ag values from 0.01 to 0.03 mg L^−1^ [57]. This value is extremely high considering that the study was carried out on infusions and not on leaves. In fact, the author of that study stated that eventual sources of contamination could not be completely excluded.

In humans, aluminum (Al) exposure via the consumption of contaminated food is associated with Alzheimer’s disease [58]. In soil, Al salts dissociate at pH < 5.5 and form complexes with phosphate that are absorbed by the root and transported to the leaves, where Al^3+^ forms strong complexes with organic acids or polyphenols that accumulate on older leaves [59]. Tea plants is known to be an Al hyperaccumulator, and older leaves may contain up to ten times more Al than younger ones, as reported for Pb and Hg. Excessive tea intake can more than double an individual’s basic Al intake [60]. In an extreme case, the Al content in old tea leaves was recorded at values exceeding 30 g kg^−1^ [61]. Hence, Al is the most represented element in European tea leaves (1986 ± 1086 mg kg^−1^) and teas from other countries, as demonstrated by several studies [16,17,20,23]. It is known that Al is present in higher concentrations in tea leaves with respect to other herbal plants, which rarely show contents exceeding 1000 mg kg^−1^ [27].

Chromium (Cr) is considered both an essential and toxic element based on its chemical speciation. The formation of enzymes and ribonucleic acids, blood clotting speed, and glucuronidase activity are all facilitated by Cr(III) compounds. Additionally, Cr(III) has roles in the immune system, RNA and DNA synthesis, antioxidant activities, and the production of hormones and several vitamins [62]. Anthropogenic activities such as chromium plating, metal polishing, leather tanning, and paint manufacturing have been linked to Cr(VI), a compound that is highly toxic to biota, including plants [63]. The concentrations of this element in the studied European tea leaves (0.8 ± 0.5 mg kg^−1^) were lower than many levels cited by other studies carried out in different Asian countries [14,18,20,23]. The concentration of this element in tea leaves is not different from that in other similar plants. In medical plants, the level of Cr generally ranges between 0.1 and 11.2 mg kg^−1^, with higher values recorded in *Arbutus andrachnae* (11.5 mg kg^−1^), *Melissa officinalis* (6.46 mg kg^−1^), *Mentha x piperita* (6.28 mg kg^−1^), *Sideritis* sp. (7.79 mg kg^−1^), and *Tilia cordata* (5.08 mg kg^−1^) [27].

Nickel (Ni) is one of the most important pollutants in soil, plants, and animals [64]. This element is becoming a toxic pollutant in agricultural environments due to its increasing concentrations caused by anthropogenic activities. However, a small amount of this element is essential for plant growth [65]. The contents of Ni in our samples (10 ± 5 mg kg^−1^) were similar or higher than those of tea leaves from other countries [14,17,23] and generally higher than those in medicinal plants, where Ni content usually ranges from 0.1 to 5 mg kg^−1^, though it can reach values up to 10 mg kg^−1^ in some species (e.g., *Crataegus* and *Salix*) [27].

### 3.3. Source and Accumulation Mechanisms

The results of the PCA clearly showed that the main driver acting on the separation of samples was geographical origin, and since geographical differences involve different types of soil used for cultivation, soil chemistry is the key parameter. The importance of this variable has also been demonstrated using similar multi-parameter approaches in other studies [27,40,66]. The atmospheric transport of particulate matter is another source of contamination, though it is specific to certain elements and plays a minor role. Moreover, good treatment practices can be applied to reduce contamination by the elements whose transport is affected by atmospheric contributions, as it has been demonstrated that the simple washing of leaves with distilled water can contribute to drastic drops in the concentrations of contaminants [46].

The different fermentation processes that tea leaves undergo to produce different types of tea such as black or green seem to have weaker effects on the element components of the final products. In fact, only when tea gardens were individually considered could slight differences be observed in some cases. This effect may have mainly been due to the different fermentation procedures applied by the producers, which may involve different processes and materials. Similar results were demonstrated in a study showing that the fermentation process plays a key role in the distribution of bioactive compounds, especially polyphenols, while the contents of elements are highly dependent on where the tea leaves are harvested [25].

In addition to these considerations, the results of our correlation analysis of the studied elements indicated possible synergic (Al–Mn, Cr–Zn, Hg–Mn, and Pb–V) or antagonist (Al–Fe, As–Ni, and Cu–Hg) accumulative effects for different element pairs. While there have been no studies on the interactions between Pb–V and Cu–Hg in the tea plant nor in plants in general, other reported interactions have been investigated by different authors. A study on the interaction of Al and Mn confirmed that a supply of Al increases the levels of Mn in the roots and tops of cowpeas (*Vigna unguiculata*) [67]. The synergy between Cr and Zn has also been recorded in bryophytes and vascular plants grown on manganese carbonate slag [68]. The interaction between Hg and Mn in barley (*Hordeum vulgare*) was studied by Pathak et al. [69] and an increase in Hg content when a high concentration of Mn was present was found, as in our study. The antagonist reduction of Fe uptake was demonstrated in the presence of large amounts of Al in soil [70]. The interaction between As and Ni was studied in rice (*Oryza sativa*), and an antagonist effect similar to that found in our study was observed by the authors [71].

### 3.4. Benefits and Risk Assessment

Our results demonstrate that tea from European gardens is not affected by contamination, as the contents of potentially toxic elements were found to be low.

At present, Europe has not adopted regulatory limits for the contents of toxic elements in tea leaves. The regulation setting maximum concentrations and amending regulations only refer to leafy vegetables. However, there are regulations applied by individual nations, as shown in Table 6 [72].

The levels of potentially toxic elements detected in the processed European tea leaf samples were always below the standard limits, except for those of nickel, which were higher than the limit enforced in India (5 mg kg^−1^), and copper, which were higher than the limit enforced in Canada (2 mg kg^−1^), which is still very low compared with the other limits for the same element. It is evident that for some elements such as cadmium, copper, mercury, and lead, there are multiple regulations, while for other elements, legislation is scarce. Therefore, but a standardized regulation at the EU level is necessary for producers to promote their product and to ensure proper monitoring for consumer safety.

Tea infusion is beneficial to human health because tea contains several elements and compounds that act to regulate cellular metabolism and exert antioxidant activity. However, caffeine content, Fe-chelating activity, and high contents of Al represent three main issues that must be investigated before promoting tea for human health [82]. Although the issue of caffeine content was not addressed in this work, the risk from exposure to not only Al and Fe but also the other considered elements was assessed using the hazard quotient (HQ), which describes the ratio of potential exposure to a substance and the level at which no adverse effects are expected. The HQ level of the studied elements in European tea showed the order of Al > Mn > Ni > Cr > Pb > Zn > Fe > As > Cu > Cd > Hg > Se > V > Co > Ag. Al and Mn are the most representative elements, since their HQs significantly affect the value of the hazard index (HI) compared with the others, as also demonstrated in other studies [16,20,22]. However, the HQ values in this study were always less than 1. The mean value of the HI (0.075), which is an integrated index for exposure to different elements, was also less than 1, indicating that simultaneous exposure to the studied elements is not harmful to consumers. Different studies carried out in China and Iran found HI values of below 1 [13,16,22]. In fact, considering the intake of elements from different dietary sources, the contribution of tea to total exposure is always minimal [83]. Furthermore, it is important to note that these indices are calculated by assuming the consumption of dry leaves and not an infusion, which contains lower contents of elements. In fact, the process of infusion never releases the entire quantity of elements, but there is a certain transfer rate that mainly depends on the time of infusion and the temperature of the water [9,10,27]. For example, in a review published by Chizzola [27], Ni, Cu and Zn were considered the elements with the highest recovery rates in infusions (maximum of 87, 80.9, and 67% of the total plant material, respectively), while the rates of elements such as Cd, Cr, Fe and Mn did not exceed 60%. Therefore, we can presume that these risk indices are actually overestimated.

## 4. Materials and Methods

### 4.1. Sample Collection

**Figure 5:**
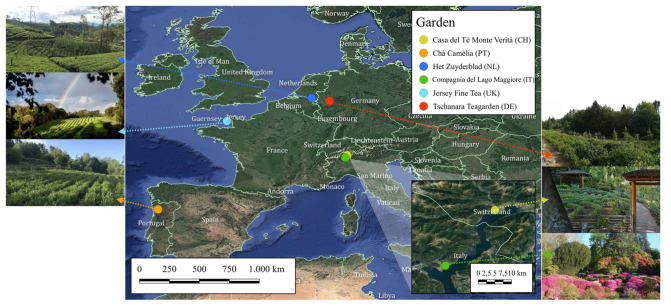
Location of European gardens where tea samples were collected. CH: Switzerland; PT: Portugal; NL: the Netherlands; IT: Italy; UK: the United Kingdom; DE: Germany.

Fresh tea leaves were hand-plucked from pruned tea bushes between May and September 2021 in six different European tea gardens: Casa del Tè Monte Verità (Ascona, Switzerland), Chá Camélia (Fornelo, Portugal), Het Zuyderblad (Soerendonk, The Netherlands), Compagnia del Lago Maggiore (Verbania, Italy), Jersey Fine Tea (Jersey, UK) and Tschanara Teagarden (Odenthal, Germany) (Figure 5). For each garden, except for Chá Camélia, two types of tea were sampled (green and black). Moreover, the Tschanara Teagarden provided samples of three black teas produced from different cultivars (the Republic of Korea, Vietnam and Azores). The samples were shipped to Italy using appropriate tea packaging and stored at room temperature until analysis.

### 4.2. Sample Treatment

All analytical steps were performed in an ISO 5 clean room laboratory. The reagents used for treatment were for trace analysis (ultrapure grade, Carlo Erba, Milan, Italy). The samples were only handled with tools that were subjected to a washing procedure using HCl 1:10 to remove metals and avoid cross-contamination. Approximately 0.5 g of each sample was introduced into PTFE liners, followed by the addition of 3 mL of HNO_3_ and 3 mL of H_2_O_2_. The liners were introduced into a microwave digester and digested using the steps indicated by Girolametti et al. [84] and reported in Table S2. In each process, one of the samples was set as a temperature control using a dedicated probe. After this step, the samples were placed in PP tubes and stabilized by adding 4 mL of ultrapure water. The samples were then stored at +4 °C and protected from light until analysis.

### 4.3. Chemical Analysis

The analysis of 14 elements (Ag, Al, As, Cd, Co, Cr, Cu, Fe, Mn, Ni, Pb, Se, V, and Zn) was carried out using the graphite furnace atomic absorption spectroscopy technique (GFAAS, 240Z AA, Agilent Technologies, Santa Clara, CA, USA) following the instrumental parameters indicated in Table S3. Readings were taken in triplicate (*n* = 3). Argon 5.0 (99.999% purity, Sol S.p.a., Ancona, Italy) was used as the carrier gas, and for some elements, the use of a matrix modifier (200 µg L^−1^ Pd in citric acid) was evaluated to optimize the quality of the signal [85,86]. The Hg content was measured with thermal decomposition amalgamation atomic absorption spectrometry (TDA AAS) using a direct mercury analyzer (DMA−1, FKV, Milestone, Sorisole, Italy) [87,88]. The analytical instrumental parameters are indicated in Table S4. The element contents were measured using calibration curves. The linearity of the method is reported in Tables S3 and S4, together with the instrumental LOD and LOQ measured for the analyzed matrix according to [89]. The accuracy of the analytical methodology used was assessed through the use of DORM–2 (Dogfish Muscle CRM, National Research Council of Canada, Ottawa, ON, Canada) and 1648a (Urban Particulate Matter SRM, National Institute of Standards and Technology, Gaithersburg, MD, USA) [90,91]. The percentage of error between the measured and certified values is shown in Tables S3 and S4.

### 4.4. Statistical Analysis

The results are expressed as mean ± standard deviation (min–max). Statistical analyses were performed using the RStudio software (R version 4.2.2) and the “ggplot2” and “corrplot” packages. Each group was compared using a one-way analysis of variance (ANOVA). Multiple comparisons were made using Tukey’s test at 95% confidence level. In order to test the homogeneity of the variance, Levene’s test was applied. In case of statistically significant differences in the variance, the Welch correction was applied to the ANOVA test. A principal component analysis (PCA) was applied to the dataset in order to investigate multivariate relationships among our variables. Significant components were obtained with the Wold cross-validation procedure [92]. The correlations between elements were evaluated using a correlation plot. For statistical analysis, V concentrations below the LOQ were considered half of the LOQ value [93].

### 4.5. Health Hazard Estimation

The potential non-carcinogenic health risk associated with element exposure through tea drinking was estimated based on the hazard quotient (HQ). An HQ of less than 1 shows no significant risk of non-carcinogenic effects on consumers. The HQ was measured by comparing the average daily intake dose (ADD) to the corresponding daily intake reference dose (RfD) (Equations (1) and (2)) [22]:

HQ = ADD/RfD(1)

ADD = C × IR/BW(2)

where C (mg kg^−1^) is the mean concentration of the element in tea, IR is the average tea consumption by Europeans (23.48 g person^−1^ day^−1^) [94], BW is the average body weight of a European adult (70 kg), and RfD (mg kg^−1^ day^−1^) is the daily intake reference dose suggested by the United States Environmental Protection Agency (USA EPA) or World Health Organization (Joint FAO/WHO Expert Committee on Food Additives). Since children rarely have the habit of drinking tea, the health risk assessment was only performed for adults [20]. The hazard index (HI) expresses the overall or interactive effects of exposure to two or more contaminants. Therefore, the total non-carcinogenic health risk of multiple contaminants was estimated as shown below (Equation (3)) [22].

HI = HQ_1_ + HQ_2_ + HQ_3_ + … + HQ_n_ (3)

## 5. Conclusions

Tea is one of the most consumed beverages in the world, and it is part of the culture and tradition of many nationalities. This study provides insights into the contents of essential and potentially toxic elements for the first time in tea leaves collected from European gardens. European tea leaves were found to contain higher levels of aluminum and nickel than more common medical plants, while the cadmium and lead contents were far lower than the same. A multivariate statistical approach was used to identify geographical origin as the main parameter that affects the contents of metals in the leaves of tea, whereas the type of processing of tea leaves was found to play a weaker role in the distribution of these elements. From the analysis of the risk indexes, it was estimated that the consumption of these products does not pose any health hazard to European consumers, but a standardized regulation at the EU level is necessary for producers to promote their product and to ensure proper monitoring for consumer safety. Further studies considering the chemical composition of soil close to the roots in order to investigate the uptake fluxes in fresh, unprocessed leaves are planned.

## Figures and Tables

**Table 1:** Essential element contents (mg kg^−1^) in European tea leaves.

Garden (Country)	Type	Co	Cu	Fe	Mn	Se	V	Zn
Jersey Fine Tea (The United Kingdom)	black	0.09 ± 0.01 ^e^	13.5 ± 0.3 ^b^	77 ± 3 ^c,d,e^	317 ± 7 ^f^	0.127 ± 0.005 ^a,b^	<0.06	46 ± 1 ^h^
	green	0.14 ± 0.002 ^f^	14.8 ± 0.4 ^c^	99 ± 4 ^g^	471 ± 5 ^h^	0.11 ± 0.02 ^a,b^	<0.06	39.7 ± 0.5 ^g^
	all	0.12 ± 0.03	14.2 ± 0.9	88 ± 16	394 ± 108	0.12 ± 0.01	<0.06	43 ± 5
Casa del Tè Monte Verità (Switzerland)	black	0.09 ± 0.01 ^e^	19.8 ± 0.3 ^g^	58 ± 3 ^a,b^	150 ± 6 ^c^	0.10 ± 0.02 ^a,b^	<0.06	33.7 ± 0.3 ^f^
	green	0.095 ± 0.005 ^e^	18.0 ± 0.1 ^e,f^	53 ± 4 ^a^	97 ± 4 ^b^	0.11 ± 0.01 ^a,b^	<0.06	26.8 ± 0.2 ^d^
	all	0.095 ± 0.0001	19 ± 1	55 ± 4	124 ± 38	0.11 ± 0.007	<0.06	30 ± 5
Compagnia del Lago Maggiore (Italy)	black	0.58 ± 0.02 ^h^	15.8 ± 0.7 ^c,d^	92 ± 3 ^fg^	226 ± 4 ^e^	0.104 ± 0.007 ^a,b^	<0.06	22.6 ± 0.2 ^a,b^
	green	0.007 ± 0.001 ^a^	20.3 ± 0.7 ^g^	83 ± 2 ^d,e,f^	330 ± 3 ^f^	0.12 ± 0.01 ^a,b^	<0.06	24.7 ± 0.1 ^c^
	all	0.3 ± 0.4	18 ± 3	88 ± 6	278 ± 73	0.111 ± 0.01	<0.06	24 ± 1
Het Zuyderblad (The Netherlands)	black	0.031 ± 0.001 ^b^	18.2 ± 0.5 ^f^	101 ± 2 ^g^	185 ± 8 ^d^	0.107 ± 0.009 ^a,b^	<0.06	30.6 ± 0.1 ^e^
	green	0.035 ± 0.001 ^b,c^	12.9 ± 0.2 ^a,b^	92 ± 2 ^f,g^	157 ± 5 ^c^	0.098 ± 0.006 ^a,b^	1.5 ± 0.2	23.7 ± 0.1 ^b,c^
	all	0.033 ± 0.003	16 ± 4	97 ± 6	171 ± 20	0.103 ± 0.006	1 ± 1	27 ± 5
Chá Camélia (Portugal)	green	0.065 ± 0.003 ^d^	15.1 ± 0.5 ^c^	87 ± 2 ^e,f^	8 ± 1 ^a^	0.14 ± 0.02 ^b^	<0.06	29.9 ± 0.2 ^e^
Tschanara Teagarden (Germany)	green	0.057 ± 0.006 ^c,d^	13 ± 0.3 ^a,b^	50 ± 2 ^a^	709 ± 9 ^m^	0.11 ± 0.03 ^a,b^	<0.06	24.3 ± 0.1 ^c^
	black (Vietnam cultivar)	0.250 ± 0.008 ^g^	16.9 ± 0.3 ^d,e^	68 ± 2 ^b,c^	535 ± 9 ^i^	0.14 ± 0.02 ^b^	<0.06	21.9 ± 0.2 ^a^
	black (Azores cultivar)	0.053 ± 0.005 ^b,c,d^	13.3 ± 0.5 ^a,b^	76 ± 9 ^c,d^	375 ± 2 ^g^	0.089 ± 0.006 ^a^	<0.06	22 ± 1 ^a,b^
	black (Korea cultivar)	0.090 ± 0.002 ^e^	12.2 ± 0.3 ^a^	49 ± 1 ^a^	628 ± 5 ^l^	0.09 ± 0.02 ^a^	<0.06	33.2 ± 0.3 ^f^
	all	0.11 ± 0.09	14 ± 2	61 ± 14	562 ± 143	0.11 ± 0.02	<0.06	25 ± 5

For each element, means bearing different letters indicate statistically significant differences (*p* < 0.05) among samples.

**Table 2:** Potentially toxic element contents (mg kg^−1^) in European tea leaves.

Garden (Country)	Type	Ag	Al	As	Cd	Cr	Hg	Ni	Pb
Jersey Fine Tea (The United Kingdom)	black	0.0081 ± 0.0001 ^c^	1648 ± 49 ^b^	0.11 ± 0.01 ^a^	0.012 ± 0.002 ^a,b^	2.08 ± 0.01 ^i^	0.0081 ± 0.0009 ^d,e^	6.4 ± 0.2 ^d^	0.47 ± 0.03 ^b,c,d^
	green	0.0074 ± 0.0001 ^b,c^	2105 ± 157 ^b,c,d^	0.10 ± 0.01 ^a^	0.016 ± 0.001 ^b,c^	1.150 ± 0.005 ^g^	0.0013 ± 0.0001 ^a^	8.9 ± 0.2 ^e^	0.35 ± 0.03 ^a,b^
	all	0.0078 ± 0.0005	1877 ± 324	0.103 ± 0.009	0.014 ± 0.003	1.6 ± 0.7	0.005 ± 0.005	8 ± 2	0.41 ± 0.08
Casa del Tè Monte Verità (Switzerland)	black	0.0069 ± 0.0001 ^a,b^	2645 ± 63 ^d^	0.091 ± 0.007 ^a^	0.029 ± 0.002 ^f,g^	0.43 ± 0.009 ^b^	0.0012 ± 0.0001 ^a^	16.8 ± 0.5 ^h^	0.41 ± 0.03 ^b,c^
	green	0.0120 ± 0.0004 ^d^	2636 ± 41 ^c,d^	0.09 ± 0.01 ^a^	0.0224 ± 0.0001 ^d,e^	0.500 ± 0.007 ^c,d^	0.0044 ± 0.0007 ^b^	16.7 ± 0.2 ^h^	0.53 ± 0.05 ^c,d^
	all	0.009 ± 0.004	2641 ± 6	0.0914 ± 0.0001	0.026 ± 0.005	0.46 ± 0.05	0.003 ± 0.002	16.72 ± 0.06	0.47 ± 0.08
Compagnia del Lago Maggiore (Italy)	black	0.0072 ± 0.0008 ^a,b^	1646 ± 64 ^b^	0.09 ± 0.01 ^a^	0.0183 ± 0.0006 ^c,d^	0.95 ± 0.01 ^f^	0.0084 ± 0.0008 ^d,e^	15 ± 1 ^g^	0.52 ± 0.02 ^c,d^
	green	0.0072 ± 0.0002 ^a,b^	2303 ± 75 ^b,c,d^	0.09 ± 0.02 ^a^	0.015 ± 0.001 ^b,c^	0.670 ± 0.009 ^e^	0.0017 ± 0.0002 ^a^	14.1 ± 0.3 ^g^	0.51 ± 0.03 ^c,d^
	all	0.00717 ± 0.00002	1975 ± 464	0.093 ± 0.003	0.016 ± 0.003	0.8 ± 0.2	0.005 ± 0.005	14.6 ± 0.8	0.519 ± 0.008
Het Zuyderblad (The Netherlands)	black	0.0073 ± 0.0001 ^b,c^	815 ± 30 ^a^	0.104 ± 0.003 ^a^	0.024 ± 0.001 ^e,f^	1.64 ± 0.03 ^h^	0.0047 ± 0.0008 ^b,c^	3.04 ± 0.06 ^b^	1.1 ± 0.1 ^e^
	green	0.0069 ± 0.0001 ^a,b^	735 ± 5 ^a^	0.100 ± 0.004 ^a^	0.061 ± 0.002 ^l^	0.69 ± 0.01 ^e^	0.0021 ± 0.0002 ^a^	5.02 ± 0.07 ^c^	1.06 ± 0.07 ^e^
	all	0.0071 ± 0.0003	775 ± 57	0.102 ± 0.003	0.04 ± 0.03	1.2 ± 0.7	0.003 ± 0.002	4 ± 1	1.064 ± 0.002
Chá Camélia (Portugal)	green	0.0064 ± 0.0001 ^a^	733 ± 8 ^a^	0.11 ± 0.03 ^a^	0.008 ± 0.0003 ^a^	0.520 ± 0.001 ^d^	0.0021 ± 0.0002 ^a^	0.77 ± 0.02 ^a^	0.48 ± 0.02 ^b,c,d^
Tschanara Teagarden (Germany)	green	0.0068 ± 0.0001 ^a,b^	4865 ± 933 ^e^	0.09 ± 0.01 ^a^	0.037 ± 0.002 ^h^	0.49 ± 0.03 ^c,d^	0.0145 ± 0.0003 ^g^	9.8 ± 0.2 ^e^	0.46 ± 0.03 ^b,c,d^
	black (Vietnam cultivar)	0.0064 ± 0.0001 ^a^	1843 ± 46 ^b,c^	0.09 ± 0.01 ^a^	0.054 ± 0.002 ^i^	0.430 ± 0.006 ^b^	0.0068 ± 0.0003 ^c,d^	12.2 ± 0.1 ^f^	0.61 ± 0.04 ^d^
	black (Azores cultivar)	0.0066 ± 0.0003 ^a,b^	1680 ± 158 ^b^	0.10 ± 0.01 ^a^	0.071 ± 0.004 ^m^	0.340 ± 0.005 ^a^	0.010 ± 0.001 ^e,f^	10.2 ± 0.1 ^e^	0.24 ± 0.02 ^a^
	black (Korea cultivar)	0.0068 ± 0.0002 ^a,b^	2167 ± 83 ^b,c,d^	0.10 ± 0.02 ^a^	0.032 ± 0.001 ^g,h^	0.47 ± 0.01 ^c^	0.012 ± 0.002 ^f^	9.9 ± 0.3 ^e^	0.42 ± 0.03 ^b,c^
	all	0.0067 ± 0.0002	2639 ± 1498	0.096 ± 0.003	0.05 ± 0.02	0.43 ± 0.07	0.011 ± 0.003	11 ± 1	0.4 ± 0.2

For each element, means bearing different letters indicate statistically significant differences (*p* < 0.05) among samples.

**Table 3:** Estimated hazard quotient (HQ) and hazard index (HI) of exposure to elements from consumption of European tea by European consumers.

Element	Jersey Fine Tea (United Kingdom)	Casa del Tè Monte Verità (Switzerland)	Compagnia del Lago Maggiore (Italy)	Het Zuyderblad (The Netherlands)	Chá Camèlia (Portugal)	Tschanara Teagarden (Germany)	All Tea Gardens
	HQ						
Ag	7.4 × 10^−6^	9.1 × 10^−6^	6.9 × 10^−6^	6.8 × 10^−6^	6.1 × 10^−6^	6.4 × 10^−6^	7.1 × 10^−6^
Al	4.5 × 10^−2^	6.3 × 10^−2^	4.7 × 10^−2^	1.9 × 10^−2^	1.8 × 10^−2^	6.3 × 10^−2^	4.8 × 10^−2^
As	2.3 × 10^−4^	2.0 × 10^−4^	2.1 × 10^−4^	2.3 × 10^−4^	2.5 × 10^−4^	2.2 × 10^−4^	2.2 × 10^−4^
Cd	6.7 × 10^−5^	1.2 × 10^−4^	7.9 × 10^−5^	2.0 × 10^−4^	3.8 × 10^−5^	2.3 × 10^−4^	1.5 × 10^−4^
Co	1.8 × 10^−5^	1.5 × 10^−5^	4.7 × 10^−5^	5.2 × 10^−6^	1.0 × 10^−5^	1.8 × 10^−5^	2.0 × 10^−5^
Cr	2.6 × 10^−3^	7.4 × 10^−4^	1.3 × 10^−3^	1.9 × 10^−3^	8.4 × 10^−4^	6.9 × 10^−4^	1.3 × 10^−3^
Cu	1.4 × 10^−4^	1.8 × 10^−4^	1.7 × 10^−4^	1.5 × 10^−4^	1.4 × 10^−4^	1.3 × 10^−4^	1.5 × 10^−4^
Fe	4.2 × 10^−4^	2.7 × 10^−4^	4.2 × 10^−4^	4.6 × 10^−4^	4.2 × 10^−4^	2.9 × 10^−4^	3.6 × 10^−4^
Hg	3.9 × 10^−5^	2.3 × 10^−5^	4.3 × 10^−5^	2.9 × 10^−5^	1.7 × 10^−5^	9.1 × 10^−5^	5.0 × 10^−5^
Mn	2.6 × 10^−2^	8.3 × 10^−3^	1.9 × 10^−2^	1,1 × 10^−2^	5.2 × 10^−4^	3.8 × 10^−2^	2.2 × 10^−2^
Ni	1.8 × 10^−3^	4.0 × 10^−3^	3.5 × 10^−3^	9.7 × 10^−4^	1.9 × 10^−4^	2.5 × 10^−3^	2.4 × 10^−3^
Pb	5.5 × 10^−4^	6.3 × 10^−4^	7.0 × 10^−4^	1.4 × 10^−3^	6.5 × 10^−4^	5.8 × 10^−4^	7.4 × 10^−4^
Se	3.8 × 10^−5^	3.5 × 10^−5^	3.5 × 10^−5^	3.3 × 10^−5^	4.6 × 10^−5^	3.4 × 10^−5^	3.6 × 10^−5^
V	5.6 × 10^−6^	5.6 × 10^−6^	5.6 × 10^−6^	1.4 × 10^−4^	5.6 × 10^−6^	5.6 × 10^−6^	2.6 × 10^−5^
Zn	6.9 × 10^−4^	4.8 × 10^−4^	3.8 × 10^−4^	4.3 × 10^−4^	4.8 × 10^−4^	4.1 × 10^−4^	4.7 × 10^−4^
	HI						
All	7.8 × 10^−2^	7.8 × 10^−2^	7.3 × 10^−2^	3.6 × 10^−2^	2.1 × 10^−2^	1.1 × 10^−1^	7.5 × 10^−2^

**Table 5:** Potentially toxic element contents in tea leaves expressed as mean ± standard deviation (min–max) in mg kg^−1^.

Garden or Market Location, Country	Type	Ag	Al	As	Cd	Cr	Hg	Ni	Pb	Reference
European Gardens	Black	0.007 ± 0.0006 (0.006–0.008)	1778 ± 560 (815–2645)	0.098 ± 0.007 (0.09–0.11)	0.03 ± 0.02 (0.012–0.071)	0.9 ± 0.7 (0.34–2.1)	0.007 ± 0.004 (0.001–0.012)	11 ± 5 (3.04–16.8)	0.5 ± 0.3 (0.24–1.07)	This study
European Gardens	Green	0.008 ± 0.002 (0.006–0.012)	2230 ± 1524 (735–4865)	0.098 ± 0.007 (0.09–0.11)	0.03 ± 0.02 (0.008–0.061)	0.7 ± 0.3 (0.49–1.15)	0.004 ± 0.005 (0.001–0.015)	9 ± 6 (0.77–16.7)	0.6 ± 0.3 (0.35–1.6)	This study
Sylhet and Moulvibazar district, Bangladesh	Black			(0.003–1.9)	(0.05–0.16)	(0.003–10.73)			(0.003–1.03)	[12]
India	Black			0.067 ± 0.036 (0.007–0.14)	0.02 ± 0.013 (0.0002–0.052)		0.01 ± 0.0049 (0.0016–0.019)		0.21 ± 0.169 (0.001–0.73)	[13]
Sri Lanka	Black			0.057 ± 0.0323 (0.0083–0.13)	0.017 ± 0.019 (0.0002–0.084)		0.0076 ± 0.0046 (0.0001–0.0019)		0.14 ± 0.109 (0.01–0.6)	[13]
Cairo, Egypt	Black				0.0 ± 0.0	6.1 ± 3.5		6.5 ± 1.4	0.4 ± 0.2	[14]
Cairo, Egypt	Green				0.09 ± 0.03	n.d.		5.73 ± 2.2	1.23 ± 0.5	[14]
Cairo, Egypt	Herbal				0.1 ± 0.06	0.5 ± 0.53		1.35 ± 0.38	0.15 ± 0.16	[14]
Local market, China	Green		487.57 ± 234.46 (227.41–911.67)		0.055 ± 0.020 (0.025–0.11)	1.63 ± 0.67 (0.28–1.63)		7.71 ± 2.91 (2.71–13.57)	0.92 ± 0.42 (0.12–2.24)	[23]
Southwest of China, China	Different varieties			(0.001–2.42)	(0.005–0.620)	(0.13–49.0)			(0.04–2.90)	[15]
Anhui, China	8 varieties		1836.77 ± 829.68 (742.81–4128.00)		0.01 ± 0.01 (n.d.–0.03)				1.07 ± 0.54 (0.23–2.59)	[16]
Gilan, Iran	Black		1143		<0.76	<1.54		10.03	1.91	[17]
India	Black		891.2		<0.68	<1.56		4.88	1.34	[17]
Ceylon	Black		968.2		<0.55	<1.21		5.09	1.71	[17]
Hsinchu market, Taiwan	Green			n.d.	n.d.	0.1 (n.d.–0.5)			0.01 (n.d.–0.2)	[18]
Hsinchu market, Taiwan	Oolong			0.005 (n.d.–0.01)	0.005 (n.d.–0.02)	5.2 (3.9–6.2)			0.4 (n.d.–1.2)	[18]
Hsinchu market, Taiwan	Black			0.01 (n.d.–0.05)	0.07 (n.d.–0.1)	7.92 (5.5–9.3)			2.01 (n.d.–6.5)	[18]
Wushwush, Ethiopia	Green				<LOD				<LOD	[19]
Guizhou, China			7323 ± 1752 (4300–10400)	0.291 ± 0.067 (0.189–0.453)	0.061 ± 0.012 (0.040–0.087)	1.47 ± 0.70 (0.69–2.91)	0.063 ± 0.015 (0.043–0.089)	9.44 ± 3.55 (3.43–14.20)	0.931 ± 0.196 (0.560–1.265)	[20]
China	Green						0.0063 ± 0.0064 (0.0018–0.103)			[21]
Yunnan, China	Puerh		1538 ± 341 (1080–2020)	0.049 ± 0.013 (0.0024–0.0066	0.003 ± 0.004 (n.d.–0.01)		0.005 ± 0.005 (n.d.–0.13)		0.94 ± 1.08 (0.31–3.42)	[22]

n.d.: not detected.

**Table 6:** Concentration of potentially toxic elements in European tea leaves vs European limit in foodstuff and other limits proposed in different regulatory frameworks and scientific contributions (mg kg^−1^).

Country or Institution	Foodstuff	As	Cd	Cr	Cu	Hg	Ni	Pb	Reference
Europe	Leaf vegetable		0.2					0.1	[11,44]
Europe	Rice	0.2							[73]
Europe	Fish					0.5			[11,44]
Australia	Tea				150				[74]
Australia	Tea				50				[75]
Canada	Raw herbal materials		0.3		2	0.2		10	[76]
China (NY/T 288–2012)	Tea				30			5	[77]
China (NY 659–2003)	Tea	2	1	5		0.3			[78]
Germany	Tea				40				[75]
Germany	Product of plant origin		0.2			0.1		5	[49]
India	Tea				150		5	10	[79]
Iran	Not reported	0.15			150	0.2		1	[74]
Japan	Tea				100				[74]
Singapore	Finished herbalproducts				150	0.5			[76]
Thailand	Tea					2		10	[74]
UK	Tea				150				[74]
USA	Tea								[74]
Vietnam	Tea and tea products		1.0			0.5		2.0	[80]
World Health Organization (WHO), 1998	Not reported		0.3					10	[81]
Measured value	Tea	0.098 ± 0.7	0.03 ± 0.2	0.8 ± 0.5	16 ± 3	0.006 ± 0.004	10 ± 5	0.6 ± 0.2	This study

## Data Availability

Not applicable.

## Supplementary Materials

The following supporting information can be downloaded at: https://www.mdpi.com/article/10.3390/molecules28093802/s1, Table S1. Principal component analysis, Eigenvalues, and explained and cumulative variance; Table S2. Mineralization operating conditions; Table S3. Instrumental parameters for element analysis of GFAAS; Table S4. Instrumental parameter for Hg analysis of TDAAAS. Figure S1. Score plot of the 5 principal components (PCs) in relation to the garden of origin (a) and treatment process (b) of European tea leaves.
