# Supplementary material for: Essential and Potentially Toxic Elements (PTEs) Content in European Tea (Camellia sinensis) Leaves: Risk Assessment for Consumers
Source: Molecules. 2023 Apr 28;28(9):3802. doi: 10.3390/molecules28093802 (PMC10179902; doi:10.3390/molecules28093802)
Supplement: Supplementary file 1 [file molecules-28-03802-s001.zip › molecules-2357515-supplementary.pdf]

molecules

*Supplementary material*

# Essential and Potentially Toxic Elements (PTEs) Content in European Tea (*Camellia sinensis*) Leaves: Risk Assessment for Consumers

**Federico Girolametti [1], Anna Annibaldi [1,*], Silvia Illuminati [1], Elisabetta Damiani [1], Patricia Carloni [2] and Cristina Truzzi [1]**

[1] Department of Life and Environmental Sciences, Università Politecnica delle Marche, 60131 Ancona, Italy; f.girolametti@staff.univpm.it (F.G

[2] Department of Agricultural, Food and Environmental Sciences, Università Politecnica delle Marche, 60131 Ancona, Italy

* Correspondence: a.annibaldi@univpm.it; Tel.: +39-071220-498

**Table S1**. Principal Component Analysis. Eigenvalues, explained and cumulative variance.

| | Principal components | | | | |
|---|---|---|---|---|---|
| | 1 | 2 | 3 | 4 | 5 |
| Variance explained | | | | | |
| Eigenvalues | 4,22289 | 2,7725 | 2,50188 | 1,37585 | 1,19619 |
| % of variance | 28.153 | 18.483 | 16.679 | 9.172 | 7.975 |
| Cumulative % | 28,153 | 46,636 | 63,315 | 72,488 | 80,462 |
| Factor loadings | | | | | |
| Ag | –0,0455593 | –0,323852 | –0,0617072 | –0,330136 | 0,482557 |
| Al | –0,388787 | –0,0713065 | 0,173521 | –0,188068 | 0,0418116 |
| As | 0,364358 | 0,149907 | 0,319427 | –0,14853 | –0,157848 |
| Cd | –0,153563 | 0,439917 | –0,256956 | –0,0746207 | –0,0128545 |
| Co | –0,102071 | –0,119786 | –0,0970032 | 0,738325 | 0,100468 |
| Cr | 0,30041 | –0,0998803 | 0,270817 | 0,195381 | 0,433567 |
| Cu | 0,0137763 | –0,45642 | –0,278423 | –0,0697878 | –0,164397 |
| Fe | 0,36775 | 0,0160961 | –0,135568 | 0,389727 | 0,0274175 |
| Hg | –0,27598 | 0,275461 | 0,300134 | 0,157204 | 0,111749 |
| Mn | –0,290169 | 0,237335 | 0,295999 | 0,108482 | –0,00480343 |
| Ni | –0,364536 | –0,288416 | –0,197713 | 0,0695249 | 0,180904 |
| Pb | 0,274368 | 0,151896 | –0,292513 | –0,0662493 | 0,188898 |
| Se | 0,151022 | –0,249045 | 0,13715 | –0,0263418 | –0,546291 |
| V | 0,164714 | 0,336907 | –0,332005 | –0,173628 | 0,221347 |
| Zn | 0,189396 | –0,164746 | 0,433572 | –0,106821 | 0,293444 |

**Table S2**. Mineralization operating conditions.

| Step | Oven Power (W) | Power (%) | Time (min) | Temperature (°C) | Hold time (min) |
|---|---|---|---|---|---|
| 1 | 800 | 100 | 10 | 150 | 5 |
| 2 | 800 | 100 | 10 | 160 | 5 |
| 3 | 800 | 100 | 10 | 175 | 5 |

**Table S3**. Instrumental parameters for elemental analysis on GFAAS.

| | Ag | Al | As | Cd | Co | Cr | Cu | Fe | Mn | Ni | Pb | Se | V | Zn |
|---|---|---|---|---|---|---|---|---|---|---|---|---|---|---|
| Wave-length (nm) | 328.1 | 396.2 | 193.7 | 228.8 | 240.7 | 357.9 | 327.4 | 248.3 | 279.5 | 232.0 | 283.3 | 196.0 | 318.5 | 213.9 |
| Furnace steps, °C (s) | | | | | | | | | | | | | | |
| 1 | 85 (5) | 85 (5) | 85 (5) | 85 (5) | 85 (5) | 85 (5) | 85 (5) | 85 (5) | 85 (5) | 85 (100) | 85 (10) | 85 (5) | 85 (5) | 85 (5) |
| 2 | 95 (30) | 95 (40) | 95 (40) | 95 (2) | 95 (40) | 95 (40) | 95 (40) | 95 (40) | 95 (40) | 95 (30) | 95 (25) | 95 (40) | 95 (40) | 95 (40) |
| 3 | 120 (10) | 120 (10) | 120 (10) | 120 (8) | 120 (10) | 120 (10) | 120 (10) | 120 (10) | 120 (8) | 120 (5) | 120 (5) | 120 (10) | 120 (10) | 120 (10) |
| 4 | 700 (25) | 1000 (5) | 1400 (5) | 550 (5) | 750 (5) | 1000 (5) | 800 (5) | 700 (5) | 700 (5) | 1200 (5) | 500 (5) | 1000 (5) | 1000 (5) | 300 (5) |
| 5 | 700 (2.9) | 1000 (1) | 1400 (1) | 50 (1) | 750 (1) | 1000 (1) | 800 (1) | 700 (1) | 700 (1) | 1200 (5) | 500 (10) | 1000 (1) | 1000 (1) | 300 (1) |
| 6 | 750 (2.9) | 1000 (2) | 1400 (2) | 550 (2) | 750 (2) | 1000 (2) | 800 (2) | 700 (2) | 700 (2) | 1200 (2) | 500 (2) | 1000 (2) | 1000 (2) | 300 (2) |
| 7 | 2100 (0.8) | 2500 (0.8) | 2600 (0.6) | 1800 (0.8) | 2300 (0.8) | 2600 (1.2) | 2300 (0.8) | 2300 (0.8) | 2400 (1.1) | 2800 (0.8) | 2100 (0.8) | 2600 (0.8) | 2700 (0.9) | 1900 (0.8) |
| 8 | 2100 (2) | 2500 (2) | 2600 (2) | 1800 (2) | 2300 (2) | 2600 (2) | 2300 (2) | 2300 (2) | 2400 (2) | 2800 (1) | 2100 (1) | 2600 (2) | 2700 (2) | 1900 (2) |
| 9 | 2150 (2) | 2500 (2) | 2600 (2) | 1800 (2) | 2300 (2) | 2600 (2) | 2300 (2) | 2300 (2) | 2400 (2) | 2800 (2) | 2700 (2) | 2600 (2) | 2700 (2) | 1900 (2) |
| Linearity, p–value (r) | 0.0014 (0.9986) | 0.0061 (0.9939) | 0.0036 (0.9964) | 0.0064 (0.9936) | 0.0002 (0.9998) | 0.0003 (0.9997) | 0.0009 (0.9991) | 0.0333 (0.9986) | 0.0000 (1.0000) | 0.0005 (0.9995) | 0.0008 (0.9992) | 0.0290 (0.9990) | 0.0624 (0.9952) | 0.0269 (0.9731) |
| LOD (μg $kg^{-1}$) | 0.69 | 50 | 11 | 0.8 | 1.1 | 3.3 | 5.3 | 10.2 | 0.08 | 2.2 | 2.8 | 6.4 | 19.5 | 1.2 |
| LOQ (μg $kg^{-1}$) | 2.08 | 152 | 33 | 3 | 3.4 | 10.1 | 16.1 | 30.9 | 0.25 | 6.7 | 8.6 | 19.3 | 59 | 3.8 |
| Accuracy, % | 2 | 17 | 14 | 0.04 | 5 | 10 | 10 | 9 | 2 | 10 | 1 | 1 | 6 | 5 |

**Table S4**. Instrumental parameter for Hg analysis on TDAAAS.

| Wavelength, nm | 253.7 |
|---|---|
| Linearity, p–value (r) | 0.0001 (0.9999) |
| LOD, μg $kg^{-1}$ | 0.6 |
| LOQ, μg $kg^{-1}$ | 1.9 |
| Accuracy, % | 2 |

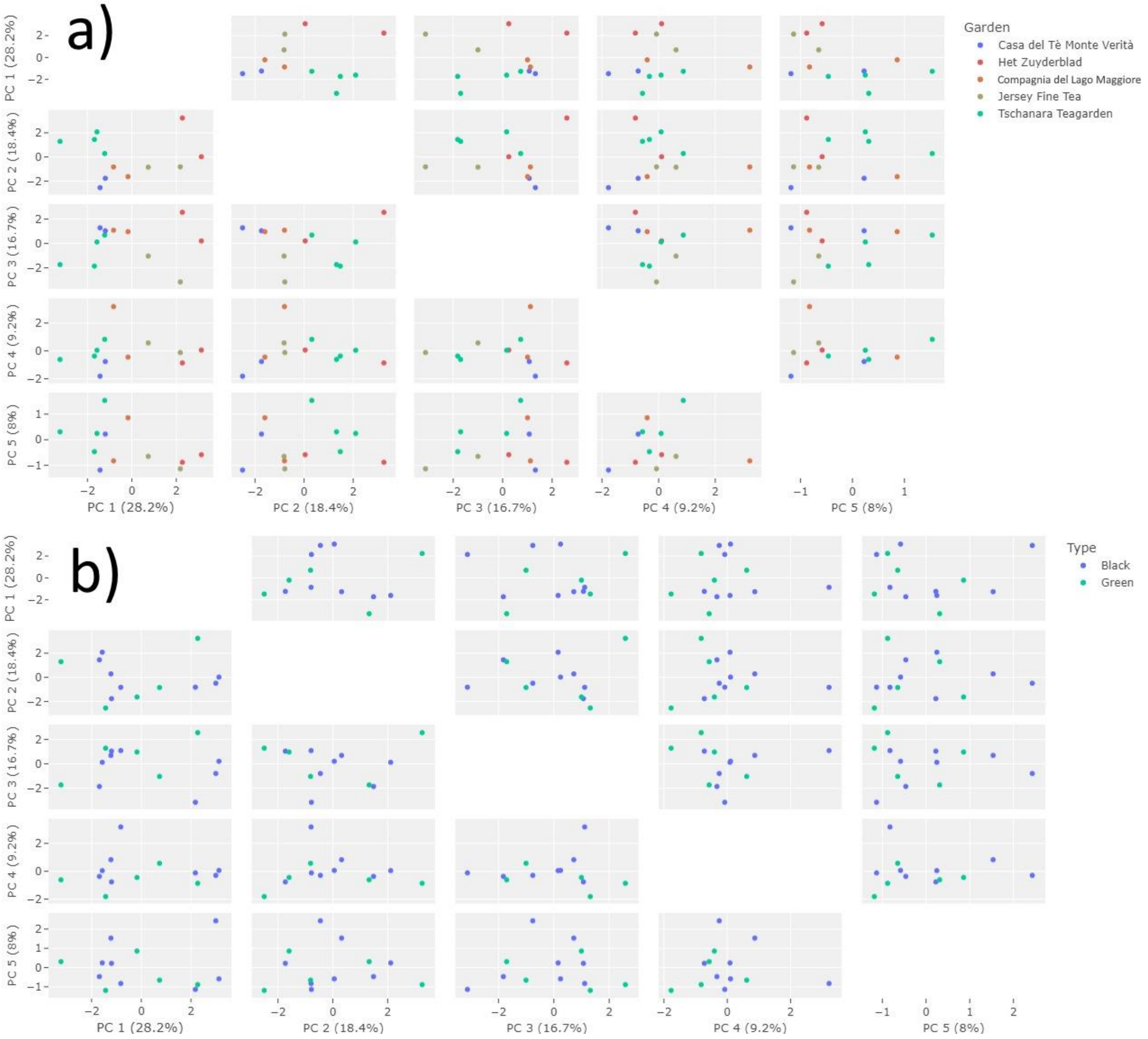


**Figure S1**. Score plot of the 5 Principal Components (PCs) in relation to the garden of origin (**a**) and treatment process (**b**) of European tea leaves.
